# Rare Occurrence of Simultaneous Dissection, Perforation and Thrombosis of External Iliac Artery Following Diagnostic Coronary Angiography: A Case Report

**Published:** 2010

**Authors:** Younes Nozary, Omid Hashemi Fard

**Affiliations:** 1 Interventionist, Tehran University of Medical Sciences, Tehran, Iran; 2 Interventionist, Isfahan Cardiovascular Research Center, Isfahan, Iran

**Keywords:** Perforation, Dissection, Thrombosis, External iliac artery, Acute arterial occlusion

## Abstract

Lower extremity complications are the most common problems encountered during transfemoral diagnostic coronary artery angiography. Dissection, thrombosis and perforation of arteries of lower extremities although not uncommon, very rarely occur simultaneously. We did not find any report in the literature in this issue. In this report we describe a case of simultaneous occurrence of all three complications during coronary angiography in one patient. It also represents some of our uncertainties regarding the best management of the patient. In this patient, arterial perforation and dissection was managed conservatively, but we applied an invasive treatment (surgical thrombectomy) for arterial thrombosis with excellent short and long-term results.

## Introduction

Local vascular complications are amongst the most common complications of diagnostic coronary angiography. Perforation and dissection should be suspected when the patient complains of acute pain at the moment of guidewire or catheter manipulation. Contrast injection may confirm extravasations of blood.^1^ For catheter perforation symptoms are usually dramatic and rapid in onset and include acute pain and hypotension. Catheter withdrawal may paradoxically lead to more discomfort as extravasations increases when the defect becomes exposed. Bleeding from guidewire perforation may be less obvious since the defect is small and extravasations is slow. Treatment is usually expectant but in case of ongoing alteration in vital signs and decline in hemoglobin, surgical exploration can be done. Effective catheter-based treatment includes an ipsilateral approach (or contralateral one if the problem is low in the iliacs) for localization and tamponade of the retroperitoneal bleeding site using a peripheral angioplasty balloon followed by placement of a covered stent as possible alternatives.^2^ This is particularly relevant when the cause is a sheath induced laceration of a tortuous iliac artery bleeding from which can be fatal within a matter of minutes without such catheter-based control.^3^ The key to preventing perforation is to observe the entire path of guidewire and catheter advancement by fluoroscopy at all times and to always lead with the guidewire. Tactile feedback is important. When encountering resistance to advancement, the operator should withdraw, redirect or otherwise ensure that the wire or catheter is in correct position.^4^ In contrast to perforation and dissection which can be managed conservatively, thrombosis is usually progressive and requires urgent intervention like thrombectomy by fogarty catheter (as in this case) or thrombosis extraction by rheolytic devices.^5^

## Case report

**Figure 1:**
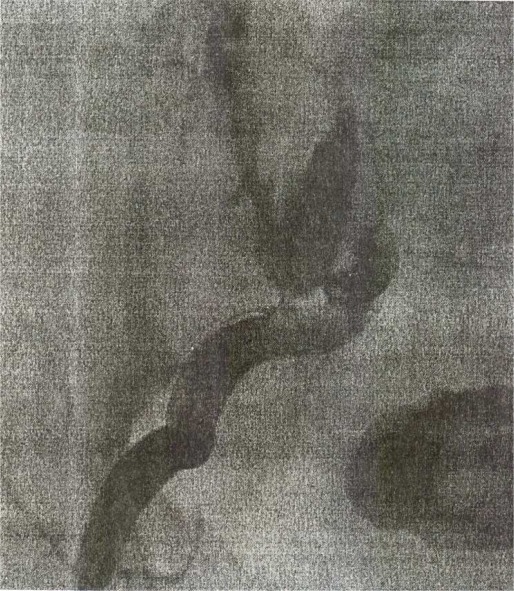
Tiny dissection and perforation of external iliac artery.

A 67 year-old male patient was referred for coronary angiography because of intermittent episodes of chest pain accompanied by severe sweating during which ECG showed transient LBBB. Physical examination was unremarkable and lab tests were within normal limits except borderline diabetes mellitus for which no specific treatment had been given. ECG at stable patient condition was normal and echocardiography showed no abnormality at rest. After prep and drape, 7F sheath was inserted in right femoral artery. Left coronary angiography showed significant stenosis of LAD midportion. Selection of RCA was impossible due to severe tortousity of right external iliac artery and kinking of right Judkin's catheter. So a 135 cm, 0.035 inch guidewire was inserted, deformed catheter was removed and replaced by another right Judkin's catheter. But advancement of guidewire and the second Judkin's catheter was difficult and the patient had extreme discomfort, so sheath angiogram was performed (figure 1).

**Figure 2:**
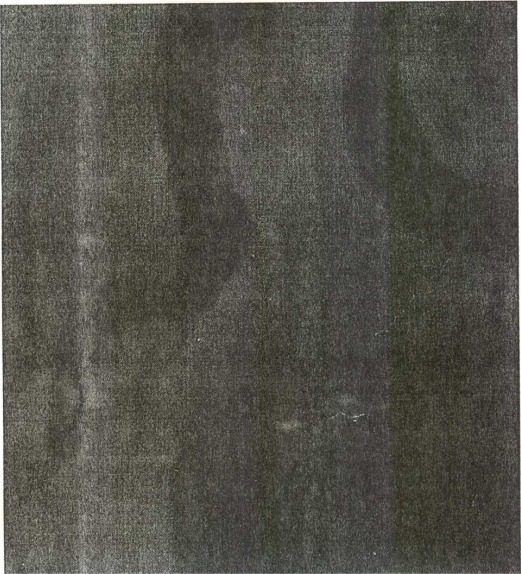
Final appearance of external iliac artery

Right iliac artery dissection and perforation was diagnosed, procedure was prematurely terminated, sheath was removed and firm compression was applied to puncture site for about 30 minutes. Several minutes after release of compression, the patient developed severe right leg pain which was accompanied by coldness and absence of pulse in distal portion. Duplex sonography confirmed weak arterial impulse in right leg and patient was transferred emergently to operating room with the impression of acute arterial occlusion. Arteriotomy was done and several pieces of arterial clots were extracted by Fogarty catheter. A small hole which was attributed to arterial puncture and sheath insertion was primarily repaired in common femoral artery. No other site of perforation was found in external iliac artery exploration. The patient had an uneventful post-operative course except wound infection which was managed successfully by IV antibiotics and debridement. Two weeks later, PCI was done on LAD via left femoral artery. RCA angiography showed no significant stenosis. Abdominal aortography was done and external iliac artery revealed to be patent and free of disease (figure 2).

## Disscussion

In this case we had three not uncommon peripheral vascular complications of coronary angiography (i.e., dissection, perforation and thrombosis of external iliac artery); occurrence of all three in one patient is quite rare. First of all, it seems that forceful guidewire advancement which was probably already in subintimal layer was a key factor in extending dissection and producing perforation. This was aggravated by sheath advancement and not properly situated guidewire.^6^ Perforations which are produced by 0.035 inch guidewires are quite small and negligible and redirection of guidewire will place it in the proper position, but when this tiny perforations are extended by sheath introduction, severe extravasations of blood occurs. This underscores gentle guidewire advancement and confirmation of proper guidewire position before introduction of sheath. We managed these complications conservatively as many of dissections of lower limb arteries following coronary angiography do not extend and do not impair limb blood flow and many of perforations respond simply to interventions like prolonged compression. During this conservative approach, the patient developed symptoms of acute arterial occlusion which was confirmed by duplex sonography. Prolonged compression of arterial puncture site (which was originally done to control bleeding of arterial perforation) was a contributing factor for development of thrombosis. Moreover, the patient had a tiny dissection in external iliac artery which works as a nidus for formation of thrombosis. We had to apply an invasive treatment for this complication because of devastating symptoms of acute arterial occlusion and progressive nature of arterial thrombosis. In surgical exploration, there was no evidence for external iliac artery perforation and the tiny hole in common femoral artery was attributed to access site puncture (so our conservative approach for arterial perforation had worked). Also, several pieces of arterial clots were extracted which confirmed clinical and sonographic diagnosis of acute arterial occlusion. In the postoperative period, anticoagulation was discontinued. Although heparin therapy seems logical after a thrombotic event, fear of ongoing bleeding from an unsuspected perforation made us withhold anticoagulant in this patient which was under close observation for recurrence of symptoms of acute limb ischemia.
